# Transgenerational inheritance of susceptibility to diabetes-induced male subfertility

**DOI:** 10.1038/s41598-017-05286-0

**Published:** 2017-07-10

**Authors:** Gabriela Pavlinkova, Hasmik Margaryan, Eva Zatecka, Eliska Valaskova, Fatima Elzeinova, Alena Kubatova, Romana Bohuslavova, Jana Peknicova

**Affiliations:** 1 Laboratory of Molecular Pathogenetics, Institute of Biotechnology CAS, BIOCEV, Vestec, Czechia; 2 Laboratory of Reproductive Biology, Institute of Biotechnology CAS, BIOCEV, Vestec, Czechia

## Abstract

Male infertility is a worldwide problem associated with genetic background, environmental factors, and diseases. One of the suspected contributing factors to male infertility is diabetes mellitus. We investigated the molecular and morphological changes in sperms and testicular tissue of diabetic males. The study was performed in streptozotocin-induced type 1 diabetes mouse model. Diabetes decreased sperm concentration and viability and increased sperm apoptosis. Changes in protamine 1/protamine 2 ratio indicated reduced sperm quality. The testicular tissue of diabetic males showed significant tissue damage, disruption of meiotic progression, and changes in the expression of genes encoding proteins important for spermiogenesis. Paternal diabetes altered sperm quality and expression pattern in the testes in offspring of two subsequent generations. Our study revealed that paternal diabetes increased susceptibility to infertility in offspring through gametic alternations. Our data also provide a mechanistic basis for transgenerational inheritance of diabetes-associated pathologies since protamines may be involved in epigenetic regulations.

## Introduction

Male fertility disorders are the primary or contributing cause of over half of all cases of infertile couples and they are instigated by a number of factors, such as genetic background, environmental factors, and diseases^1^. One of the suspected factors contributing to male infertility is diabetes mellitus (DM). DM as a risk factor of male reproduction has been recognized only recently. For many years, the relationship between DM and abnormalities of male reproductive function has been controversial and inconclusive^2, 3^. The prevailing views that DM has little effect on male fertility have been based on routine semen analysis. However, more sensitive analytical techniques have shown that DM induces subtle molecular changes, which negatively affect spermatogenesis, sperm quality and function, and penile erection and ejaculation^4, 5^. Clinical data from *in vitro* fertilization clinics show that pregnancy rates are significantly lower for diabetic male patients, suggesting that diabetes-exposed sperms are damaged^6, 7^. However, the mechanisms responsible for male fertility disorders in association with DM are not established.

Besides the direct adverse effects of the diabetic environment on the reproductive system and reproductive outcomes, long-term complications in offspring exposed to the maternal diabetic intrauterine environment have been recognized^8–11^. Increasing evidence indicates that paternal environmental exposures also affect offspring phenotype. For example, paternal obesity affects the hypomethylation of insulin-like growth factor in human newborns^12^, pre-mating fasting of male mice affects serum glucose levels in offspring^13^, high fat diet exposure of male rats reprograms ß cells in offspring^14^, and offspring of mouse males fed a low-protein diet show changes in liver expression profiles^15^. Paternal prediabetes increases the susceptibility to diabetes in offspring through altered methylation patterns in sperm, involving changes in methylation of insulin signaling genes^16^. These results characterize the mechanistic basis for the transgenerational inheritance of susceptibility to diabetes via male germ cells.

Male germ cells undergo extensive and unique chromatin and epigenetic remodeling during spermatogenesis. During meiosis and mitosis, the DNA of male germ cells is packaged in nucleosomes, comprised of histones, which are covalently modified during spermatogenesis (for review see ref. 17). During the elongating spermatid stage, most histones are replaced with protamines, small basic proteins that bind DNA and produce tightly packed structures, important for sperm maturation. Many reports have shown that the protamine 1/protamine 2 ratios (P1/P2) are important for sperm quality and DNA stability in humans^18–21^ as well as mice^22^. Protamines may also play a role in paternal genome imprinting and in the establishment of epigenetic marks that can be transmitted to the oocyte upon fertilization and thus influence the embryo^20, 23–25^. Moreover, some regulatory elements escape systematic DNA demethylation in primordial germ cells, providing an additional basis for transgenerational epigenetic inheritance^26^. Thus, altered histone modifications, DNA methylation, and improper histone to protamine replacement in sperm may affect early embryogenesis and increase susceptibility to complex multifactorial diseases and disorders, such as DM and infertility in the offspring.

The goal of this study was to provide a complex analysis of the molecular and morphological changes in the testes and sperms induced by diabetes. For the first time, we showed the transgenerational inheritance of adverse effects of paternal diabetes on the reproductive system of offspring in an STZ-induced diabetes model.

## Results

### Changes in physiological and biochemical parameters after 6 weeks of diabetes

**Figure 1:**
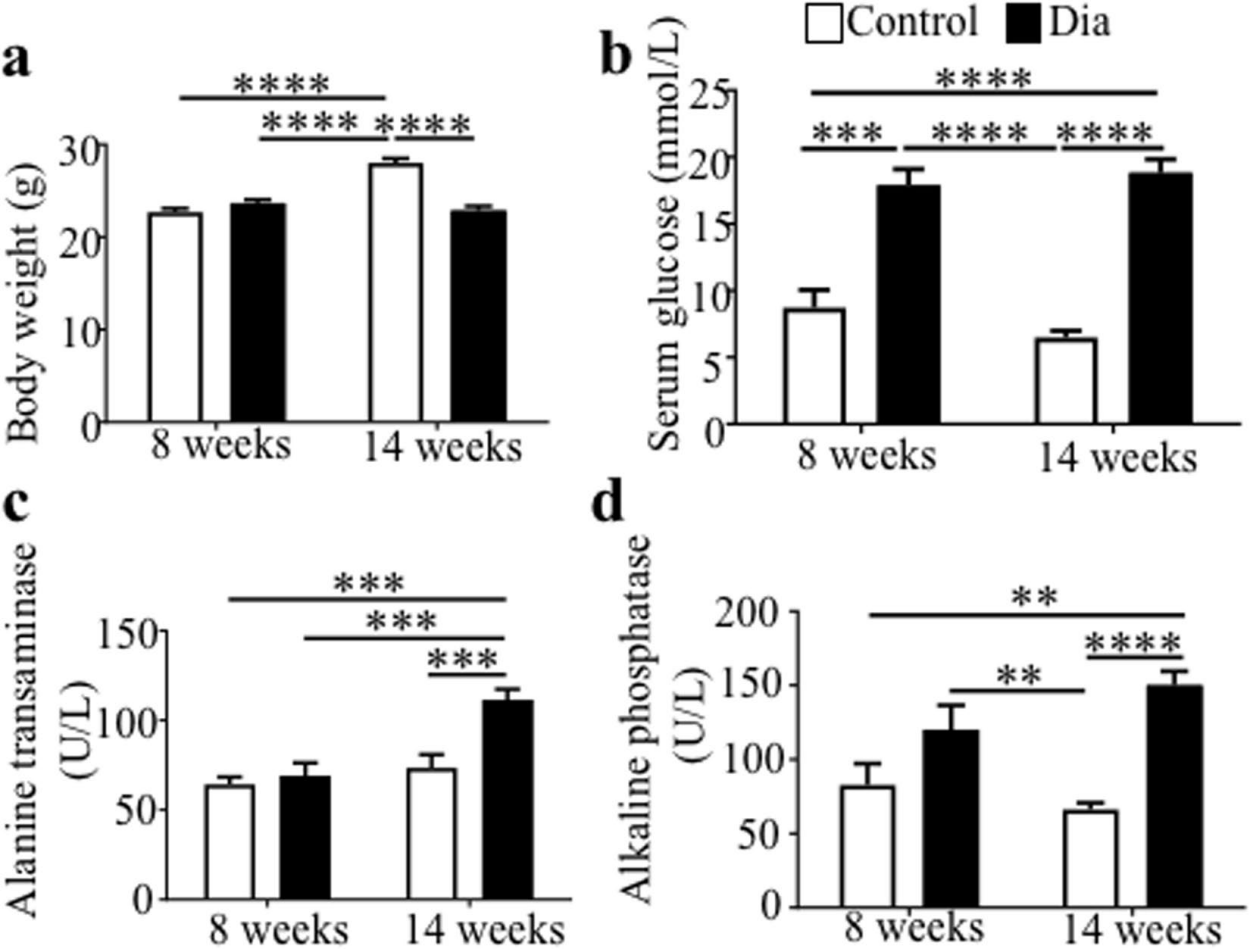
Changes in body weight and serum biochemical characteristics at the start of the experiment (8 weeks of age) and at the end of the experiment (14 weeks of age). The body weight and serum biochemical parameters were measured at the beginning of the study and after 6 weeks of diabetes-exposure. (**a**) body weight and (**b**) serum level of glucose, blood glucose levels maintained above 13.9 mmol/L are classified as diabetic. (**c**) Changes in the serum levels of alanine transaminase and (**d**) alkaline phosphatase. The values represent means ± SEM, (n = 12/group). **P < 0.01, ***P < 0.001, ****P < 0.0001 compared all pairs by Tukey’s post-test.

For this study, we used the well-established low-dose STZ-induced diabetes mouse model on the FVB genetic background^27–29^. Body weight was reduced and the weights of the kidney and liver were increased in diabetic groups compared to non-diabetic, control mice (Fig. 1a, Supplementary Table S3). The weight of reproductive organs, epididymis and seminal vesicles, was decreased in diabetic mice. The anogenital distance (AGD), as an androgen-responsive outcome, was not affected (Supplementary Table S3). The levels of fasting glucose and selected enzymes were significantly different between control and diabetic mice over the 6-week study (Fig. 1b–d).

### Effects of diabetic exposure on sperm parameters of diabetic males and transgenerational transmission of the phenotype to offspring generations

**Figure 2:**
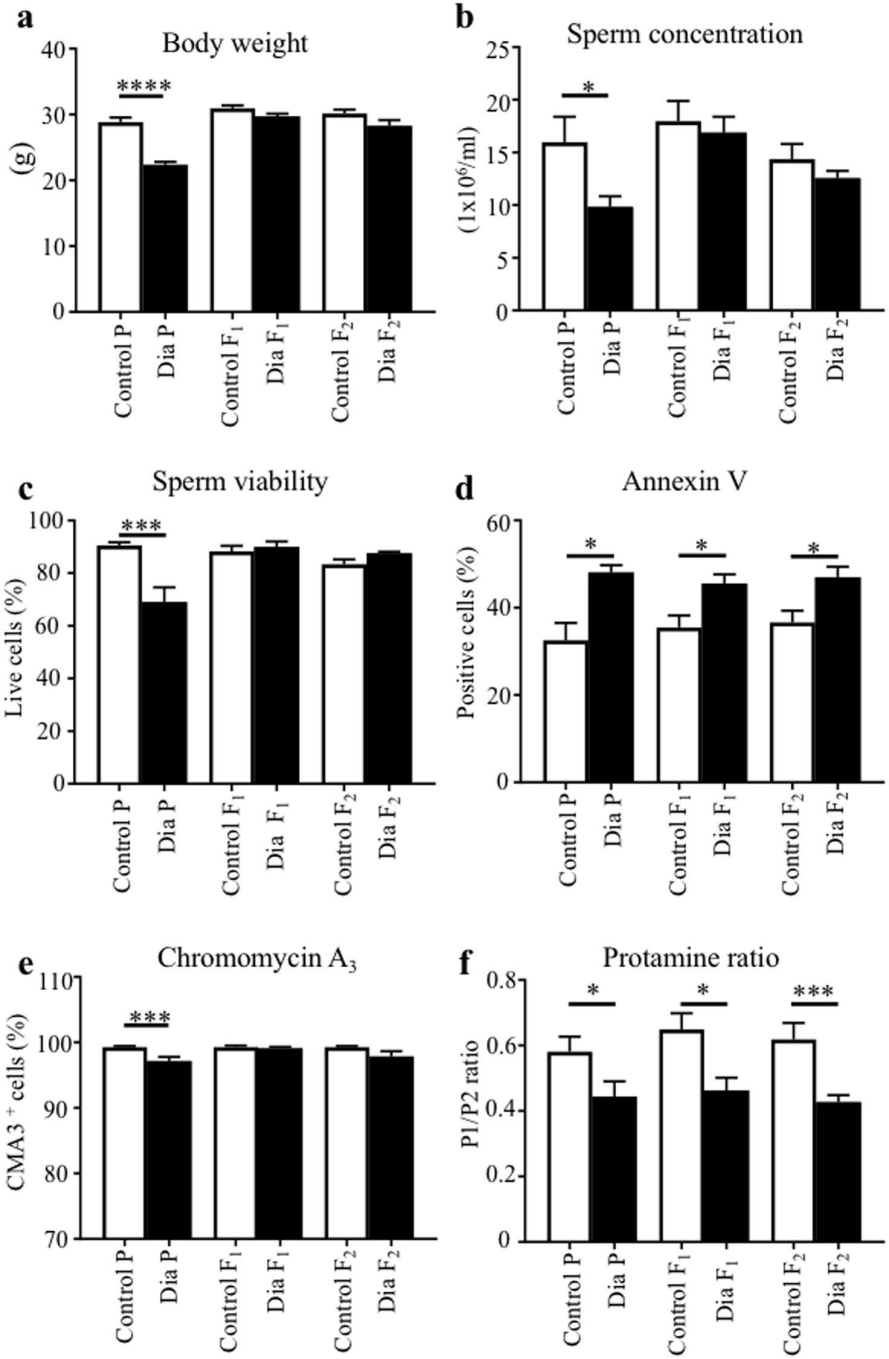
Transgenerational phenotype transmitted from diabetic father (P) through the male germ line. Effects of diabetes on body weight (**a**), sperm concentration (**b**), sperm viability (**c**), the level of apoptotic marker annexin V (**d**), percentage of chromomycin A_3_ positivity for chromatin packaging evaluation (**e**), and (**f**) protamine ratios were determined in caudal epididymal sperms from diabetic and control non-diabetic males (P) and from subsequent F_1_ and F_2_ male progeny. Results are expressed as the mean ± SEM (Control P = 14, Dia P = 13, Control F_1_ = 15, Dia F_1_ = 37, Dia F_2_ = 20). *P < 0.05, ***P < 0.001, ****P < 0.0001 *vs*. Control group for each generation by Dunnett’s post hoc tests.

We analyzed sperm parameters in diabetic males (parental generation, P) and the subsequent effects of diabetic exposure on sperm parameters of the offspring of diabetic males in F_1_ and F_2_ generations. Caudal epididymal sperm were collected for a sperm quality assessment. Sperm concentration and viability was reduced by 34% and 27%, respectively, for diabetic males compared to controls, whereas the F_1_ and F_2_ offspring demonstrated no apparent effects (Fig. 2b,c). The increased level of apoptotic marker annexin V was detected in epididymal sperm samples from diabetic males (Fig. 2d). Interestingly, subsequent F_1_ and F_2_ male progeny had an increase in sperm cell apoptosis, as indicated by annexin V compared to the controls (Fig. 2d). The sperm head morphology and percentage of separated sperm heads were comparable between diabetic and non-diabetic males (data not shown). To assess the packaging quality of the chromatin^30^, chromomycin A_3_ staining of sperms was performed. Changes in chromomycin A_3_ staining were detected only in the parental generation of diabetic mice (Fig. 2e). To further assess sperm quality and sperm DNA packaging, we analyzed protamine 1 and protamine 2 ratios. Strikingly, the protamine 1 and protamine 2 ratios were altered not only in the parental diabetic generation but also in the subsequent F_1_ and F_2_ offspring generations (Fig. 2f). Our assessments of semen parameters indicate adverse effects of the diabetic environment on sperm quality. However, the reproductive performance of diabetic males was similar to controls. All diabetic males were able to mate in the period of one week and produce comparable litter size (8.1 ± 0.9, n = 12 litters), as controls (8.4 ± 1.2, n = 8 litters). The litter size was not affected in any of the groups of the offspring (data not shown).

### Histological evaluation of the diabetic testes shows two phenotypes

**Figure 3:**
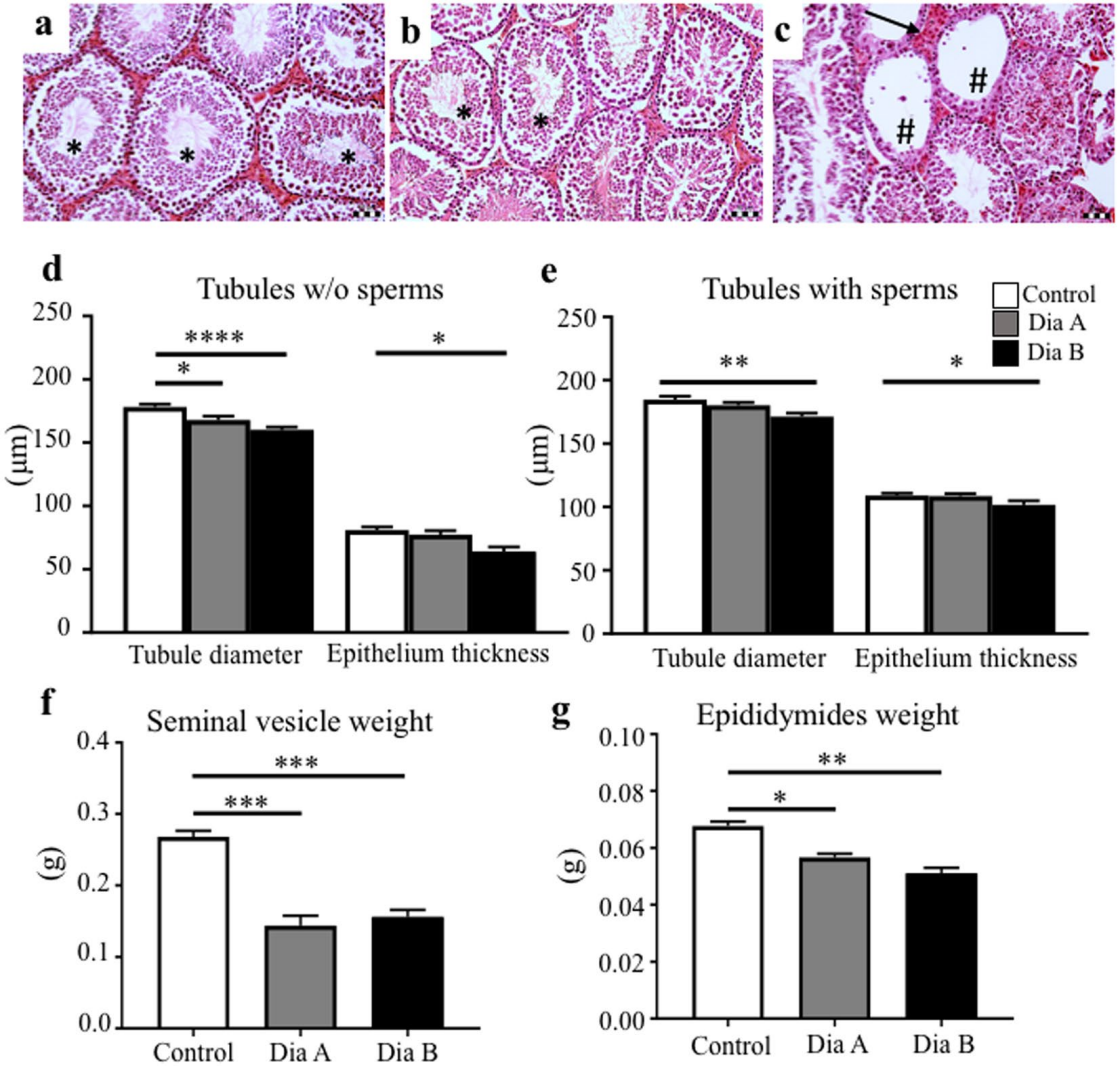
Testicular morphology evaluation. Representative images for a control (**a**) and diabetic (**b**,**c**) testis sections after H &E staining show the germinal epithelium lining seminiferous tubules in various stages of spermatogenesis. Based on changes in testicular histology, we separated diabetic males into two groups, the first diabetic group with less evident changes (**b**) and a second diabetic group with more severe destruction of the seminiferous epithelium (**c**), including a thinner and disrupted epithelium, and atrophy of the seminiferous tubules with a loss of germ cell populations (as marked with #). Asterisks indicate the presence of spermatozoa in the seminiferous tubules. The interstitial tissue with an expansion of amorphous material indicated by arrow. Scale bar 50 μm. (**d**,**e**) The tubule diameter and epithelium thickness were evaluated in controls (n = 14) and diabetic group with less manifested histological changes (Dia A, n = 7) and group B with more severe morphological defects (Dia B, n = 5). For each individual, 60 tubules with sperm cells and 60 tubules without sperm cells were evaluated. (**f**,**g**) Both Dia A and Dia B groups have a significant reduction of seminal vesicle and epididymides weight compared to controls. The values represent means ± SEM. *P < 0.05, **P < 0.01, ***P < 0.001 *vs*. Control group by Dunnett’s post hoc tests.

The histology of control testes showed smoothly rounded seminiferous tubules lined by germinal epithelium at various stages of spermatogenesis (Fig. 3a). In contrast, in the testes of diabetic mice, a disrupted and thinner germinal epithelium was noticeable, as shown in a representative Fig. 3c. Nevertheless, all the distinct developmental stages of spermatogenesis, including spermatozoa in the lumen of the seminiferous tubules were identified. However, we and others^31, 32^ observed high individual variations in testicular histology within the diabetic group. Some seminiferous tubules showed spermatocytes and early spermatids which did not form regular “columns”, or premature sloughing of spermatocytes or seminiferous tubules were anthropic without any cells in the tubule lumen (Fig. 3c, pound sign). Additionally, the interstitial tissue of diabetic mice seemed less compact with an expansion of amorphous material compared to control testes (arrow in Fig. 3c). As the thickness of the seminiferous epithelium is influenced by the presence or absence of releasing spermatozoa, we separately evaluated the seminiferous tubules containing sperms and the seminiferous tubules without sperms. Based on the severity of changes in testicular histology, diabetic males were separated into two groups, the first diabetic group (Dia A) with morphological abnormalities in less than 20% of evaluated seminiferous tubules (Fig. 3b) and a second group of diabetics (Dia B) with more significant destruction of the seminiferous epithelium, including testicular atrophy (Fig. 3c). The tubular diameter was significantly reduced in both diabetic groups compared to controls, whereas the thickness of the epithelium was significantly reduced only in the Dia B group, correlating with more disrupted testicular histology (Fig. 3d,e). Correspondingly to unfavorable testicular histology, after 6 weeks of diabetes, diabetic males showed a reduction in seminal vesicle and epididymides weights (Fig. 3f,g).

Based on the severity of changes in testicular histology, the parental diabetic male generation was divided into two groups and their offspring were analyzed separately. The body weight was significantly changed in the F_1_ offspring of diabetic males with more severe changes in testicular histology (Supplementary Table S3). The transmission of this phenotype was not detected for the F_2_ generation. We also evaluated testicular histology of the F_1_ and F_2_ offspring; however, we did not identify any evident morphological changes between the offspring of diabetic and control males.

### Disruption of spermiogenesis in the testis of diabetic males

**Figure 4:**
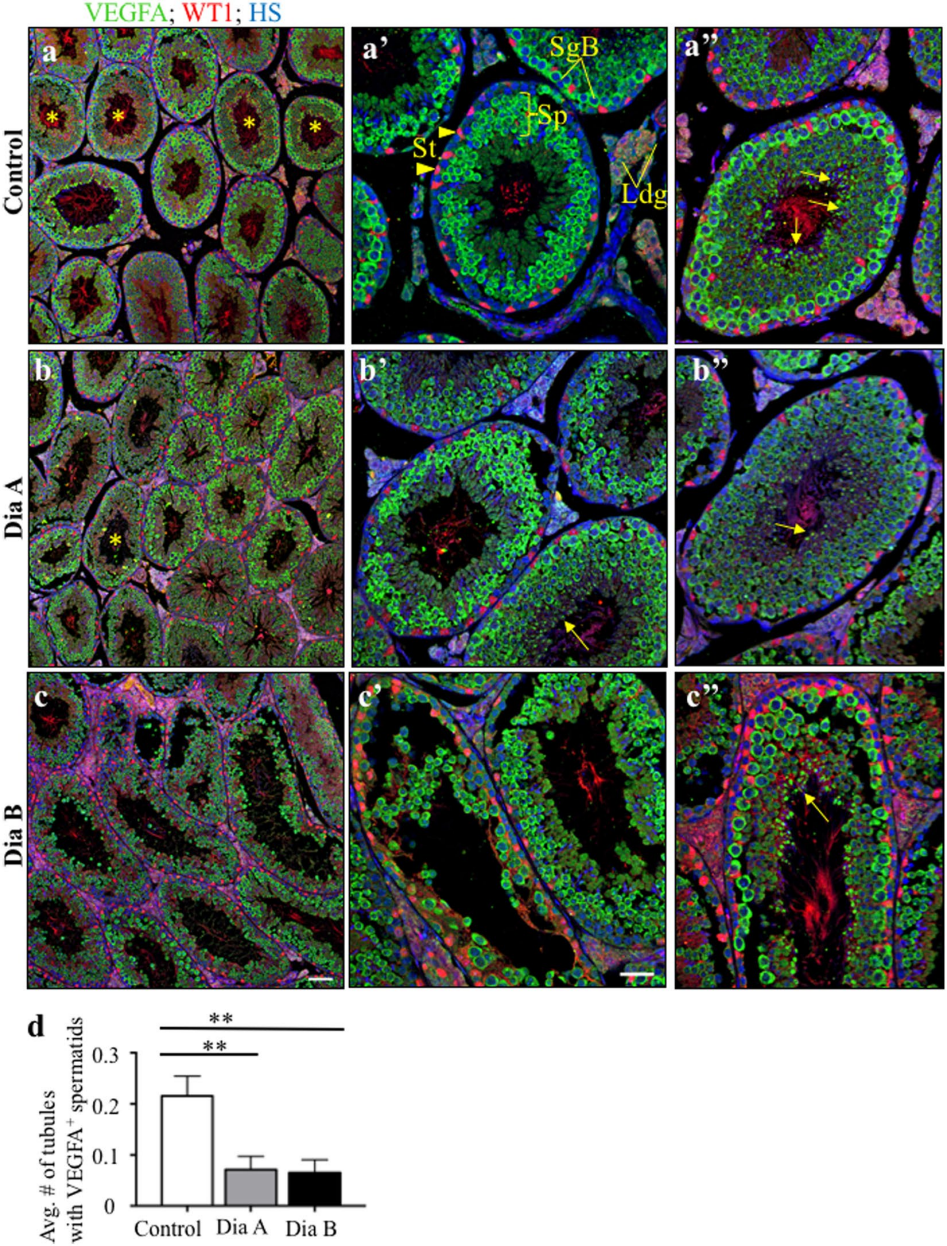
The number of the seminiferous tubules containing VEGFA^+^ spermatids was reduced in the diabetic mice. (**a**) The representative images show the expression of VEGFA (green) and WT1 (red), a marker of Sertoli cells, in various stages of spermatogenesis, the seminiferous tubules with VEGFA^+^ spermatids indicated by asterisks. (**a’**) Detail of the seminiferous tubule with strong VEGFA expression in type B spermatogonia and primary spermatocytes and (**a”**) the tubule with VEGFA^+^ spermatids (arrows). (**b**–**b”**) Representative images of the expression patterns of VEGFA and WT1 in the testes of diabetic group A (Dia A). (**c**–**c”**) The disruption of the epithelium in the seminiferous tubules is apparent in the testes of diabetic group B (Dia B), although the tubules with VEGFA^+^ spermatids are present (arrow). Nuclei are counterstained with Hoechst 33342 (blue). Scale bar 100 μm (**a**,**b**,**c**) and 50 μm (**a’**–**c”**). (**d**) The average number of the seminiferous tubules with VEGFA^+^ spermatids. The values represent means ± SEM (*n* = 3 individuals/6 sections/137 tubules/control group; 209 tubules/Dia A; 157 tubules/Dia B). **P < 0.01 *vs*. Control by Dunnett’s post hoc tests. St, Sertoli cells; SgB spermatogonia type B; Sp, primary spermatocytes; Ldg, interstitial Leydig cells

We further analyzed the morphology of the seminiferous epithelia and multiple stages of spermatogenesis. We used VEGFA as a molecular marker for type B spermatogonia to evaluate mitosis and the production of type B spermatogonia, primary spermatocytes and spermatids^33^. Various stages of spermatogenesis, including spermatozoa in the lumen of the seminiferous tubules, were identified in all experimental groups (Fig. 4). The overall expression pattern of VEGFA showed comparable testicular morphology between control and Dia A groups (Fig. 4a,b). The VEGFA^+^ spermatogonia and primary spermatocytes were detected in the testes with a severe destruction of the seminiferous epithelium (Fig. 4c). Interestingly, the number of seminiferous tubules containing VEGFA^+^ spermatids was reduced to 7% in the Dia A group compared to 21% in control mice (Fig. 4d), suggesting changes in the spermatogenesis process. The seminiferous tubules with VEGFA^+^ spermatids were found in the testes of the Dia B group, although damage to the epithelium was apparent (Fig. 4c”). Decreased production of spermatids in the diabetic testis corresponds with decreased sperm concentration in the diabetic group compared to controls (Fig. 2b). Sertoli cells were present without any detectable changes in their distribution or number even in the seminiferous tubules with a severely damaged epithelium, as shown by WT1 expression (Fig. 4c–c”).

**Figure 5:**
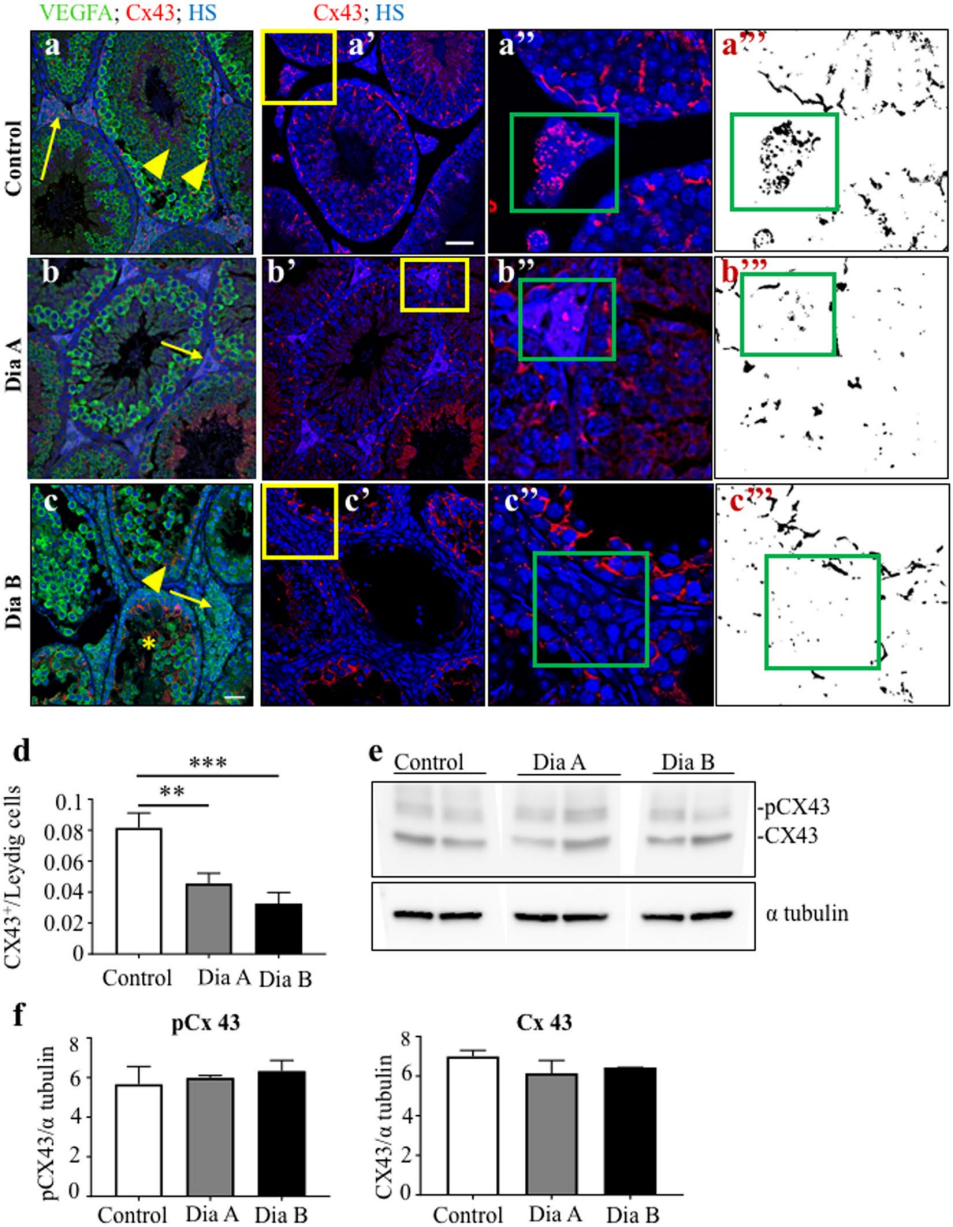
Cx43 expression in the testes of diabetic mice. Representative Cx43 staining of 8 μm testis sections of the control testes (**a**); a strong Cx43 expression (red) is between Sertoli cells and between the apical part of spermatogonia and basal to primary spermatocytes (arrowheads). Type B spermatogonia and primary spermatocytes have a strong VEGFA expression (green). Cx43 is expressed in the clusters of Leydig cells (arrows). B: Cx43 expression pattern in Dia A is comparable to controls. (**c**) Increased Cx43 expression is detectable in the damaged seminiferous tubules with a high loss of cells in the diabetic testes (asterisks). (**a’**–**c’**) Representative images of Cx43 expression (red). (**a”**–**c”**) A detail of the interstitial area with Leyding cells. (**a”’**–**c”’**) Delineated Cx43^+^ area in the testis section by ImageJ program. Nuclei are counterstained with Hoechst 33342 (blue). Scale bar 50 μm. (**d**) A relative quantification of staining determined as a ratio of Cx43^+^ areas per the total Leydig cell areas by ImageJ. The values represent means ± SEM (n = 3 individuals/3 sections/31 areas/control group; 49 areas/Dia A group; 39 areas/Dia B group). **P < 0.01, ***P < 0.0001 *vs*. Control by Dunnett’s post hoc tests. (**e**) Representative Western blot of Cx43 and phosphorylated form pCx43 with α tubulin loading controls in lysates of the testes of control and diabetic groups. (**f**) Quantitation of Cx43 and pCx43 Western blots relative to α tubulin. The values represent means ± SEM (n = 4 mice/group). Cx43, connexin 43; pCx43, phosphorylated connexin 43.

The expression of connexin 43 (Cx43) was visualized in the testes of experimental mice using immunohistochemistry^34^. Widespread Cx43 expression was detected in both diabetic and control testes with a strong Cx43 signal between Sertoli cells, spermatogonia, or primary spermatocytes (Fig. 5a–c). A noticeable expansion of Cx43 expression was revealed in the damaged seminiferous tubules with a substantial loss of cells (asterisks, Fig. 5c). Cx43 expression was also detected in the interstitial tissue containing Leydig cells (Fig. 5a’–c”’). A relative quantification of Cx43 expression demonstrated reduced Cx43 staining between neighboring Leydig cells in the diabetic testes (Fig. 5d), indicating impairment in intercellular communications. However, there was no obvious change in the overall distribution of Cx43 and its phosphorylated form in the seminiferous tubules between the control and diabetic testes, as measured by Western blotting (Fig. 5e,f).

### Progression of meiosis is impaired in diabetic males

**Figure 6:**
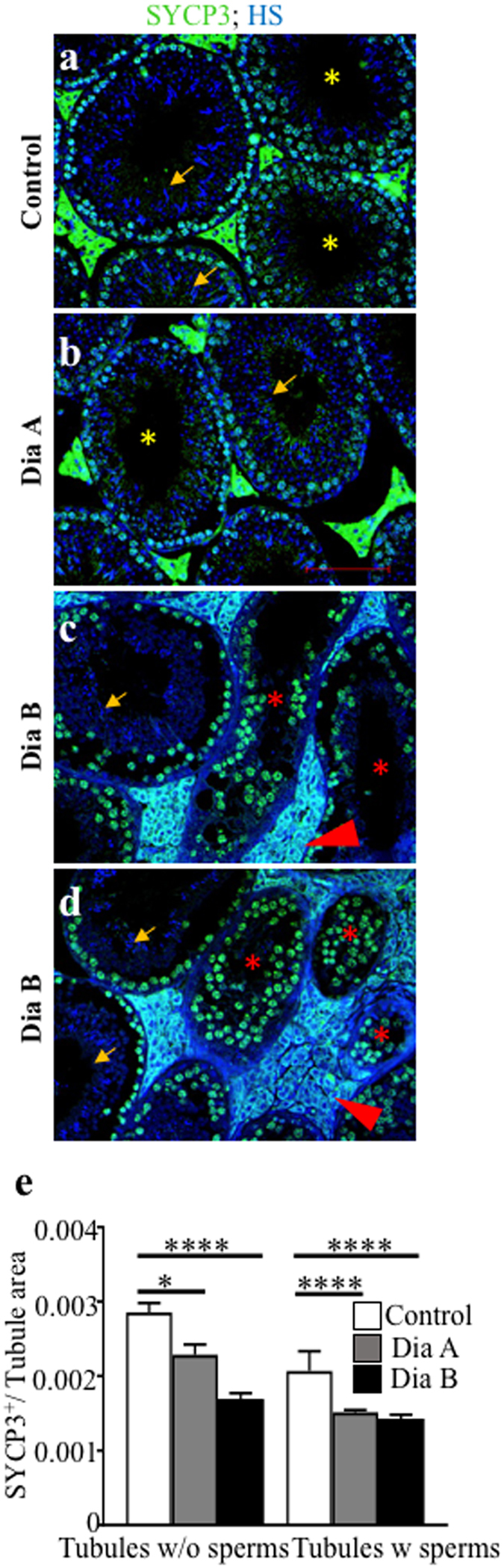
Decreased number of meiotic cells in diabetic testes as detected by SYCP3 marker. The representative images show the expression of SYCP3 (green) in various stages of spermatogenesis (**a**–**d**), nuclei are counterstained with DAPI (blue). Seminiferous tubules with mature spermatozoa (arrows) and without mature spermatozoa (yellow asterisks) are indicated. Abnormal accumulation of SYCP3^+^ spermatocytes in the seminiferous tubules of diabetic males is indicated by red asterisks. An expansion of amorphous material in the interstitial tissue is shown by red arrowheads. Scale bar 50 μm. (**e**) A relative quantification using ImageJ program determines SYCP3^+^ cell area per tubule area. Tubules with and without mature spermatozoa are evaluated separately. The values represent means ± SEM (*n* = 3 individuals/3 sections/31 areas/control group; 49 areas/Dia A group; 39 areas/Dia B group). *P < 0.05, ****P < 0.0001 *vs*. Control by Dunnett’s post hoc tests.

We analyzed the progression of meiosis and localization patterns of meiotic marker SYCP3, an axial element of the synaptonemal complex^35^. A similar pattern of SYCP3 expression was in the seminiferous tubules of males of the Dia A group compared to controls (Fig. 6a,b). In contrast, we observed an abnormal multiple layer accumulation of intense SYCP3 stained spermatocytes and the absence of postmeiotic cells in Dia B tubules (red asterisks, Fig. 6c,d), indicating changes in meiotic progression. Furthermore, the relative quantification of SYCP3 expression showed overall decreased SYCP3 expression in both Dia A and Dia B groups compared to the non-diabetic testes (Fig. 6e).

### Molecular changes in the diabetes-exposed testes of the parental generation and transgenerational transmission of changes to the F_1_ and F_2_ generations

**Figure 7:**
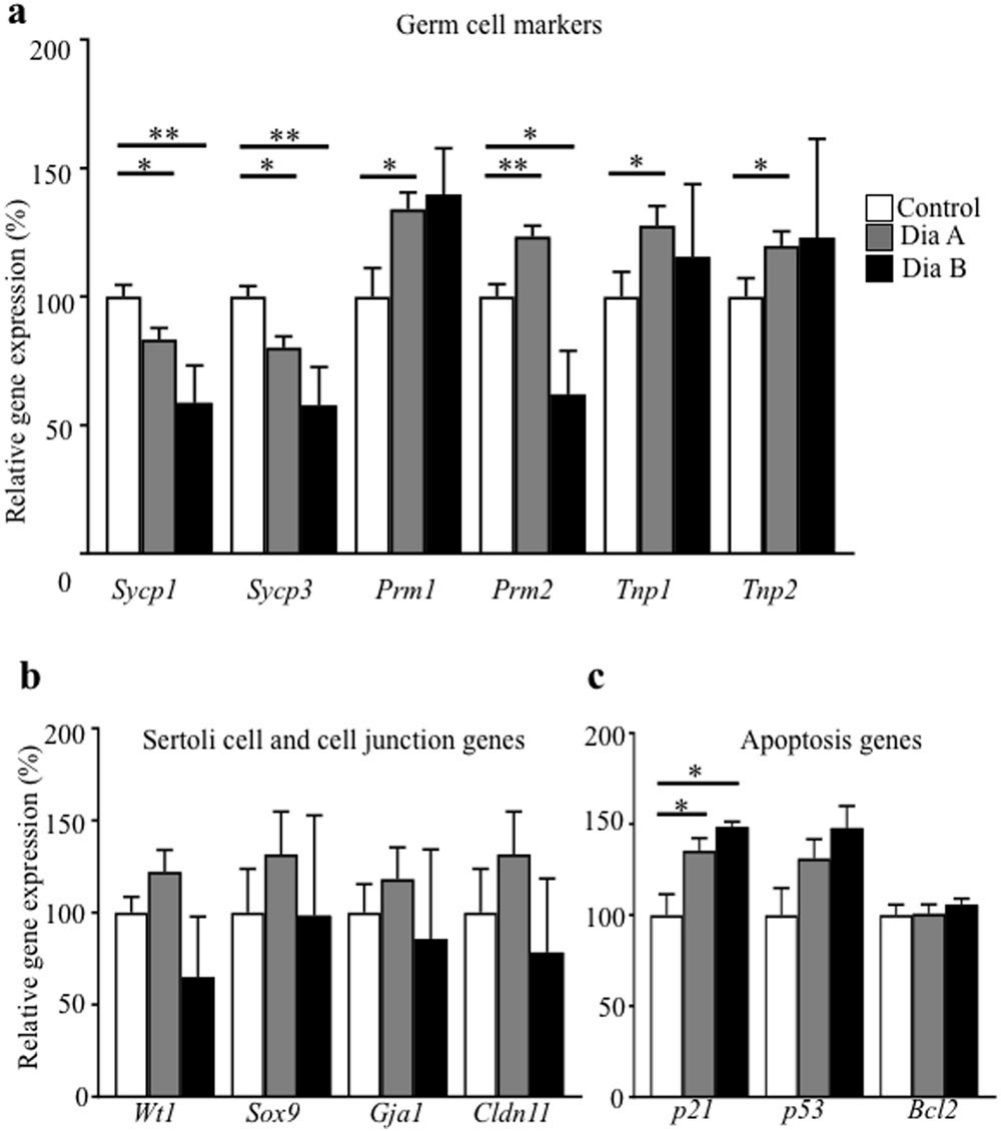
Gene expression changes in the testes in the parental generation. (**a**) qPCR analysis of markers for spermatogenesis and spermiogenesis: synaptonemal complex protein 1 (*Sycp1*), synaptonemal complex protein 3 (*Sycp3*), protamine 1 (*Prm1*), protamine 2 (*Prm1*), transition protein 1 (*Tnp1*) and transition protein 2 (*Tnp2*); (**b**) genes expressed in Sertoli cells: Wilms’ tumour 1 (*Wt1*), SRY-box containing gene9 (*Sox9*), gap junction protein alpha 1 (*Gja1*) and claudin 11 (*Cldn11*); and (**c**) apoptotic markers: cyclin-dependent kinase inhibitor 1 A (*p21*), tumor protein p53 (*p53*), and B cell leukemia/lymphoma 2 (*Bcl2*). The control group represents 100% of relative gene expression. The values are means ± SEM. Control group n = 9; Dia A group n = 8; Dia B group n = 4. *P < 0.05, **P < 0.01 *vs*. Control by Dunnett’s post hoc tests.

To assess the effects of the diabetic environment on gene expression in testicular tissue, we analyzed the expression of genes encoding markers for spermatocytes, synaptonemal complex protein 1 (*Sycp1*) and protein 3 (*Sycp3*), and for spermatids, protamine 1 (*Prm1*), protamine 2 (*Prm1*), transition protein 1 (*Tnp1*), and transition protein 2 (*Tnp2*). We also analyzed the expression of genes associated with Sertoli cell structure and/or function: Wilms tumour 1 (*Wt1*), SRY-box containing gene9 (*Sox9*), gap junction protein alpha 1 (*Gja1*) and Claudin 11 (*Cldn11*). The expression levels of gene encoding proteins involved in apoptosis (*p21, p53, Bcl-2*) were evaluated. Based on our qPCR analyses, spermatogenesis as well as spermiogenesis processes were affected by diabetes (Fig. 7a). The expression of *Tnp1*, *Tnp2, Prm1*, and *Prm2* was significantly affected in the testes of diabetic mice. We did not detect any changes in Sertoli cell and cell junction genes (Fig. 7b). However, the expression of apoptotic cascade marker *p21* was increased in diabetic mice and correlated with the adverse effects of diabetes on the testes (Fig. 7c).

**Figure 8:**
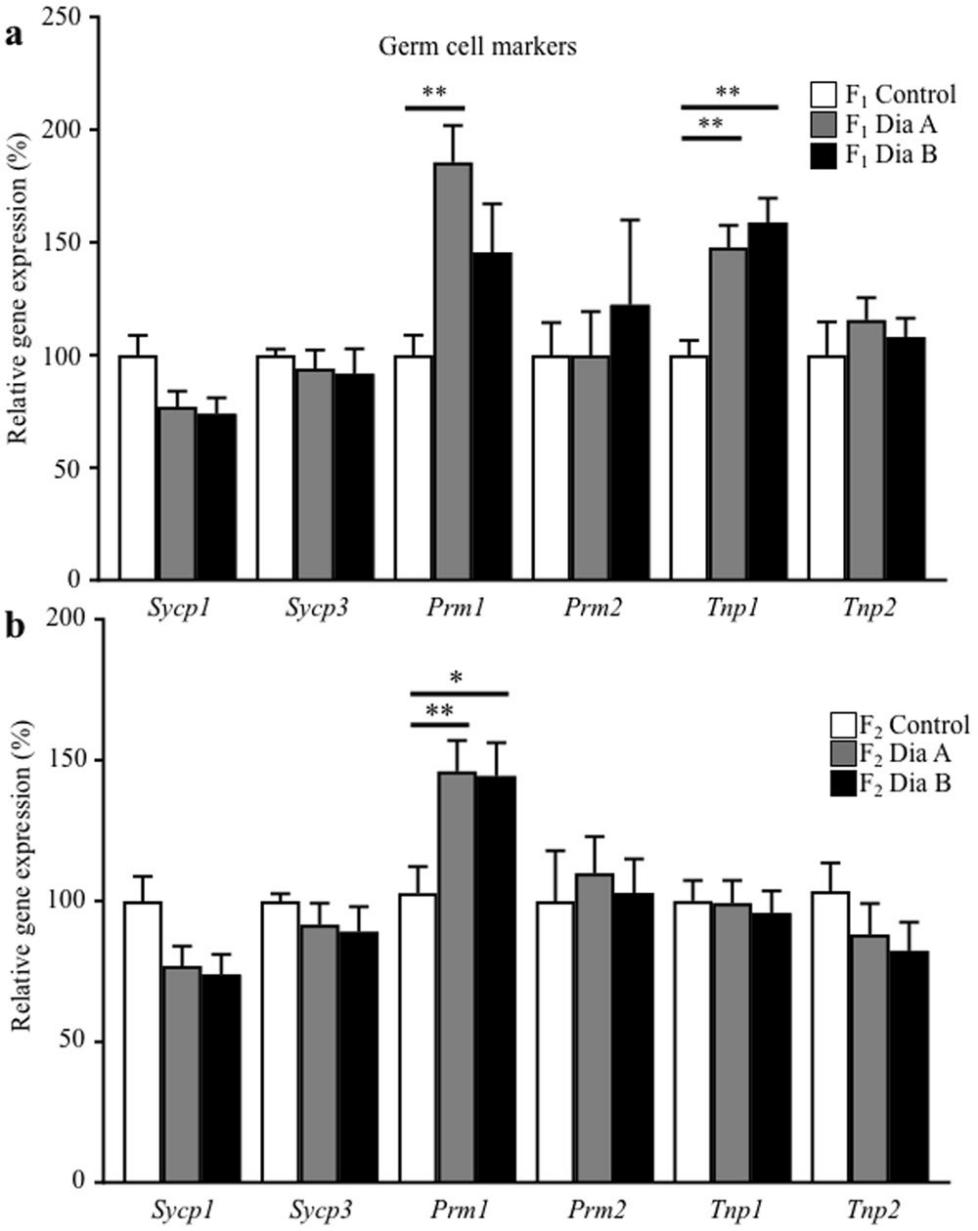
Transgenerational effects of diabetes on testicular gene expression of the F_1_ and F_2_ male generations. (**a**) qPCR analysis of the expression of genes encoding proteins associated with spermatogenesis (synaptonemal complex protein 1 (*Sycp1*), synaptonemal complex protein 3 (*Sycp3*)), and spermiogenesis (protamine 1 (*Prm1*), protamine 2 (*Prm1*), transition protein 1 (*Tnp1*) and transition protein 2 (*Tnp2*)) in the F_1_ testes (F_1_ Control, n = 8; F_1_ Dia A n = 10; F_1_ Dia B n = 5). (**b**) Expression changes in the testes of the F_2_ males (F_2_ Control n = 6; F_2_ Dia A n = 12; F_2_ Dia B n = 11). The graph illustrates the relative expression of the selected genes. The control group represents 100% of relative gene expression. The values are means ± SEM. *P < 0.05, **P < 0.01 *vs*. Control group by Dunnett’s post hoc tests.

The transmission of gene expression changes in the testicular tissues was determined for the offspring of F_1_ and F_2_ generations (Fig. 8 and Supplementary Fig. 1). No significant changes were detected in the expression of genes encoding proteins associated with Sertoli cells, gap junctions, and apoptosis (Supplementary Fig. 1). However, the F_1_ male offspring of diabetic fathers showed significant changes in the expression of genes *Prm1* and *Tnp1*, associated with the process of spermiogenesis, the markers of condensed and condensing spermatids, respectively (Fig. 8a). Altered *Prm1* expression was further transmitted to the F_2_ male generation (Fig. 8b). Changes in protamine expression correlate with significant changes in the sperm nuclear protamine protein ratios detected in the F_1_ and F_2_ male generations of diabetic fathers (Fig. 2f). These data of altered protamine levels indicate the transgenerational effects of the diabetic environment in the male germ line and thus, an increased susceptibility to spermiogenesis defects in diabetic males and subsequent male generations.

## Discussion

For the first time, our data provide a complex analysis of the effects of diabetes on the male reproductive system in type 1 diabetes model. We have shown that the diabetic environment affects sperm viability, increases sperm apoptosis, and alters sperm protamine 1/protamine 2 ratios, indicating reduced sperm quality. Additionally, the testicular tissue of diabetic mice display significant tissue damage and altered expression pattern compared to non-diabetic males. We have also demonstrated that paternal diabetes induces unfavorable changes in the subsequent F_1_ and F_2_ offspring generations. Specifically, an increase in the expression of apoptosis marker annexin V and altered protamine ratios in sperms were detected. Changes in the sperm nuclear protamine ratios correlate with the changes in the expression of protamine1 in the testes of the F_1_ and F_2_ male generations of diabetic fathers. All together our results provide evidence that the diabetic environment alters spermiogenesis and sperm quality and that this phenotype is heritable.

We used experimentally STZ-induced diabetes animal model. STZ is recognized by glucose transporter GLUT2, and mainly targeted to pancreatic β cells^36^. The half-life of STZ is extremely short between 5–15 min *in vivo* and therefore cannot function for a prolonged time. Since STZ is an alkylating agent, a number of studies have analyzed if identified epigenetic alternations were diabetes specific or the results of STZ treatment^16, 37, 38^. All these studies concluded that epigenenetic changes were a function of hyperglycemia and reduced insulin production in STZ-induced diabetes and not by STZ treatment alone.

Spermatogenesis starts with the mitotic proliferation and differentiation of spermatogonial stem cells into primary spermatocytes, followed by meiosis and production of haploid secondary spermatocytes (for review see ref. 39). The earliest postmeiotic cells produced from secondary spermatocytes are round spermatids, which turn into elongating spermatids and finally they mature into spermatozoa. After completion of meiosis, most histones are replaced by transition proteins (TP1 and TP2, encoded by *Tnp1* and *Tnp2*, respectively) and subsequently replaced by protamine 1 and protamine 2 for chromatin compaction. Our data demonstrate that the last stage of spermatogenesis, spermiogenesis, is affected by the diabetic environment. Completion of meiosis depends on the formation of the synaptonemal complex, including recruitment of SYCP1 and SYCP3 proteins. The deletion of *Sycp3* results in a disruption of meiotic progression during spermatogenesis and no development of postmeiotic cells in the seminiferous tubules of mutant testis^35^. We detected a substantial decreased expression of both *Sycp1* and *Sycp3* mRNA in the testes of diabetic mice. Accordingly, our analyses show a significantly lower expression of SYCP3 protein and less tubules with spermatids in the testes of diabetic males, indicating a disruption of meiotic development. We also detected altered expression of genes encoding transition proteins and protamines in the diabetic testes that are crucial for the completion of spermigenesis. Elevated or diminished protamine levels are linked to lower sperm counts and viability, and sperm DNA damage in humans^18–21^ and in mice^22, 40^. Correspondingly, we found altered protamine ratio, decreased sperm concentration and viability, and increased levels of apoptotic marker annexin V in the epididymal sperms of diabetic males (Fig. 2). Abnormal protamine expression is an intriguing pathology due its relationship with altered spermiogenesis and infertility. Protamines are not only important for condensation of sperm chromatin and protection of the paternal genome but protamines may be significant in reprograming of the imprinting pattern of the gamete, and thus, in epigenetic regulation^41^.

For the first time, we showed that paternal diabetes induced specific transgenerational changes in the testes and sperms of the offspring across two generations. Specifically, the testis expression of protamines, protamine ratios and level of apoptotic marker annexin V in sperms were affected in the paternal generation and in two generations of the offspring (F_1_ and F_2_ generations). These transgenerational effects of paternal diabetes indicate changes in sperm epigenetic information. In contrast, increased chromomycin A_3_ staining, indicating poorly packed sperm chromatin and protamine deficiency, was detected only in the sperms of diabetic males (parental generation). Similarly, a reduced sperm concentration and sperm viability were found only in the diabetic males. Based on our data, we can postulate that the reduced sperm concentration, sperm viability, and protamine deficiency resulted from the direct exposure to the adverse diabetic environment. Another direct impact of adverse diabetic environment was the damage of the testicular tissue of diabetic males. Since these changes were not transmitted from the paternal generation to the offspring, they may not be associated with modifications of the sperm epigenome.

Although sperm concentration and sperm quality was significantly reduced in diabetic males, we still found spermatids even in the testes with severe damage to the testicular epithelium. Correspondingly, in this stage of diabetes-induced testicular tissue damage, all diabetic males were able to mate and produce litter sizes comparable to control non-diabetic males.

It is of great interest to determine the transgenerational phenotypes associated with a direct exposure of parental generations to diabetes. Potential mechanisms contributing to transgenerational transmission of phenotypes are complex and may differ depending on whether the effects are transmitted through the maternal or paternal lineage. So far, the majority of studies have been focused on maternal effects. The maternal line of transmission of metabolic phenotype is multifactorial, including genetic effects (nuclear and mitochondrial DNA), epigenetic factors, altered placental function, and diabetic intrauterine and early postnatal environment. There is substantial clinical and experimental evidence showing that altered maternal metabolism and altered body composition of the mother (diabetes, malnutrition, obesity, unbalanced maternal nutrition) produce changes in metabolism and growth (macrosomia or growth restriction) of the offspring (for review see refs 42, 43). However, maternal effects are difficult to separate from direct effects of *in utero* environmental exposure on the offspring and environment-induced modifications in germline with multigenerational inheritance effects. On the contrary, paternal effects induced by the altered environment represent predominantly epigenetic modifications in sperm, although particular environmental factors can affect the composition of seminal fluid, which can produce placental and developmental effects^43^. A number of recent studies show epigenetic transgenerational transmission of metabolic phenotypes from males to their offspring^14–16, 44^. Environment-induced epigenetic modifications in germline cells include DNA methylation, posttranslational modification of histones and protamines, histone variants, and non-coding RNAs (for review see ref. 39).

Differences in the relative contribution of maternal or paternal effects to the transmission of metabolic phenotypes to the offspring have been reported in human studies^45–47^ as well as in experimental models^43, 44, 48, 49^. Together, clinical and experimental studies demonstrate that the exposure to adverse metabolic environment, can increase susceptibility for diabetes in the offspring and that both maternal and paternal factors contribute to offspring phenotypes.

In conclusion, we demonstrate negative effects of the diabetic environment on the male reproductive system, including morphological and expressional changes of testicular tissue, and reduction of sperm concentration and sperm quality. Furthermore, we show that paternal diabetes alters sperm quality and expression pattern in the testes in offspring of two subsequent generations. These paternally induced transgenerational non-genetic modifications of germ cells represent increased susceptibility to infertility for the next generation. Consequently, alterations in epigenetic patterns of the mature sperm in infertile males signify an increased risk for idiopathic infertility, which may be manifested in preterm birth, low birth weight, congenital anomalies, perinatal mortality, and several other pregnancy-related complications seen at a higher frequency in babies conceived by IVF^50^. It remains to be established what type of epigenetic modifications in sperms results from the perturbed testis microenvironment of diabetic males and is transmitted across two generations. However, our data are in line with other studies calling for new strategies to improve metabolic health not only in women of reproductive age but also in potential fathers in order to reduce susceptibility to disease in subsequent generations.

## Methods

### Experimental animals

This study was conducted in accordance with the Guide for the Care and Use of Laboratory Animals (NIH Publication No. 85-23, revised 1996). The experimental protocol was approved by the Animal Care and Use Committee of the Institute of Molecular Genetics, CAS and carried out in accordance with the relevant guidelines and regulations. Diabetes was induced in male inbred FVB (strain code 207, Charles River), aged 6 weeks, by intraperitoneal injection of 100 mg/kg body weight of streptozotocin (STZ; Sigma), as described^28, 29^. One week after STZ injection, blood glucose levels were measured in animals by glucometer (COUNTOUR TS, Bayer, Switzerland); blood glucose levels maintained above 13.9 mmol/L are classified as diabetic. Both control and STZ-diabetic mice were maintained without any other treatment for 6 weeks of diabetes. During this period, regular checks of glycaemia and body weight were performed. Mice were kept under standard experimental conditions with constant temperature (23–24 °C) and fed on soy-free feed (LASvendi, Germany).

### Evaluation of reproductive performance and transgenerational studies

Individual males were placed in a cage with 1 healthy adult female. The animals were kept together for a week. Immediately after separation, the males were killed, tissues and blood were collected for analyses. The females were housed individually during the gestation period and the litter size was recorded. Only male pups, the F_1_ offspring, were left in the litters to be used for follow up transgenerational studies. The F_2_ offspring were produced from the mating of randomly selected F_1_ males with control females (n = 10 males/group).

### Biochemical parameters

Blood serum was collected from non-diabetic and diabetic mice following a 6-h fast and was analyzed using a Beckman Coulter AU480 Chemistry Analyzer (Beckman) according to the manufacturer’s protocol in the Core Facility for Phenogenomics, Biocev.

### Sperm parameters

The isolation of sperms from caudal regions of the dissected epididymides was performed and sperm concentration was determined as described previously^51^. Sperm morphology (200 cells/each animal) and sperm head separation (200 cells/each animal) was evaluated using Spermac Stain kit (FertiPro, Belgium)^51^. Sperm viability was analyzed with SYBR14 from Live/Dead sperm viability kit (Molecular Probes, USA) and by flow cytometry (LSRII, blue laser 488 nm, Becton Dickinson, USA), minimum 15,000 events were evaluated. An annexin V–FITC apoptosis kit (Sigma) was used to assess the sperm damage. Samples were examined with a Nikon Eclipse E400 fluorescent microscope equipped with a Nikon Plan Apo VC 60/1.40 oil objective (Nikon Corporation Instruments Company) and photographed with a ProgRes MF CCD camera (Jenoptik, Germany) with the aid of the NIS-ELEMENTS imaging software (Laboratory Imaging, Ltd.). 200 cells were evaluated for each animal.

Chromomycin A_3_ staining was used according to the manufacture’s protocol. Briefly, 10 µL of sperm suspension on glass slide was air dried and fixed in Carnoy’s fixing solution at 4 °C for 15 min. After rinsing in Mc´Ilvaine buffer, sperms were stained with chromomycin A_3_ (CMA3, Sigma) solution. Staining intensity of a single sperm head was evaluated under fluorescence microscope Nikon Eclipse E400 and 60× oil immersion objective Plan Apo VC.

### Protamine analysis

Protamine extraction from sperm pellet (final concentration 4 × 10^6^ sperm cells) was performed as described^22^. Protamines were separated using Acid-urea polyacrylamide gel electrophoresis^52^. After electrophoretic separation, the gel was fixed in 50% methanol, 10% acetic acid (V/v) for 15 min at RT, washed in H_2_O for 15 min at RT, and stained with EZBlue gel staining reagent for 1 hour at RT. The intensity of the bands corresponding to P1 and P2 was quantified using Image J software and the P1/P2 ratio was calculated.

### Morphometric evaluation of the testis

Dissected testes were fixed with 4% paraformaldehyde in PBS (pH 7.4) at 4 °C overnight, and embedded in paraffin. Paraffin-embedded tissues were cut in 8 µm sections, de-paraffinized and rehydrated sections were stained with hematoxylin&eosin. Morphometric evaluations of seminiferous tubule diameter and thickness of the seminiferous epithelium were performed (100 tubules/sample). The diameter of a seminiferous tubule was defined as the shortest distance between two parallel tangent lines of the outer edge of the tubule using Olympus CKX4 microscope and QuickPhoto Micro 3.1 program.

### Immunohistochemical analyses

Immunohistochemistry was performed using paraffin-embedded tissue section (8 µm) with antibodies listed in Supplementary Table S1. The sections were counterstained with Hoechst 33342 (#14533 Sigma) and imaged with confocal microscope ZEISS LSM 880 NLO or with fluorescence microscope Nikon Eclipse E400. The evaluation of the number of meiotic cells per tubule diameter was done using the Adobe Photoshop CS3 program. A relative quantification of Cx43 staining in Leydig cells was done using the ImageJ program.

### Real-time reverse-transcription PCR

Total RNA was isolated from the testes using TriReagent (Sigma). Reverse transcription (RT) with 2 μg of RNA, RevertAid Reverse Transcriptase (Thermo Fisher Scientific), 1 μL Oligo(dT) and random hexamer primers (Fermentas) was performed to generate cDNA using an optimized protocol (Fermentas). Following RT, quantitative real-time PCR (qPCR) with a final concentration of cDNA 10 ng/μL was performed using a CFX 384 – qPCR cycler (BioRad). The relative expression of a target gene was calculated, based on the quantification cycle (Cq) difference (Δ) of an experimental sample *versus* control. The control was set at 100% and experimental samples were compared to the control^53^. The β-actin (*Actb*) and Peptidylprolyl isomerase A (*Ppia*) genes were used as the reference genes. We used only primers above 96% qPCR efficiency for the analysis (primer sequences are listed in Supplementary Table S2).

### Statistical analysis

The comparisons between diabetic and control mice were done using STATISTICA 7.0. (Statsoft, Czech Republic) and GraphPad Prism 7.0a (GraphPad Software, Inc., USA). Differences among diabetic A, B and control groups were tested with one-way ANOVA, follow by Tukey’s or Dunnett’s post hoc tests for multiple comparison. Differences between organ weights were tested by ANCOVA with body weight as covariate. Differences in sperm parameters were assessed by Mann–Whitney U-test. A *P* value < 0.05 was considered to be statistically significant.

## Electronic supplementary material

Supplementary data
