# Supplementary material for: Transgenerational inheritance of susceptibility to diabetes-induced male subfertility
Source: Sci Rep. 2017 Jul 10;7:4940. doi: 10.1038/s41598-017-05286-0 (PMC5504044; doi:10.1038/s41598-017-05286-0)
Supplement: Supplementary file 1 — Supplementary data [file 41598_2017_5286_MOESM1_ESM.pdf]

SUPPLEMENTARY INFORMATION

Transgenerational inheritance of susceptibility to diabetes-induced male subfertility

Gabriela Pavlinkova[1*], Hasmik Margaryan[2], Eva Zatecka[2], Eliska Valaskova[2], Fatima Elzeinova[2], Alena Kubatova[2], Romana Bohuslavova[1], Jana Peknicova[2]

[1]Laboratory of Molecular Pathogenetics, Institute of Biotechnology CAS, BIOCEV, Czechia
[2]Laboratory of Reproductive Biology, Institute of Biotechnology CAS, BIOCEV, Czechia

*Corresponding author: Gabriela Pavlinkova, gpavlinkova@ibt.cas.cz

SUPPLEMENTARY FIGURE

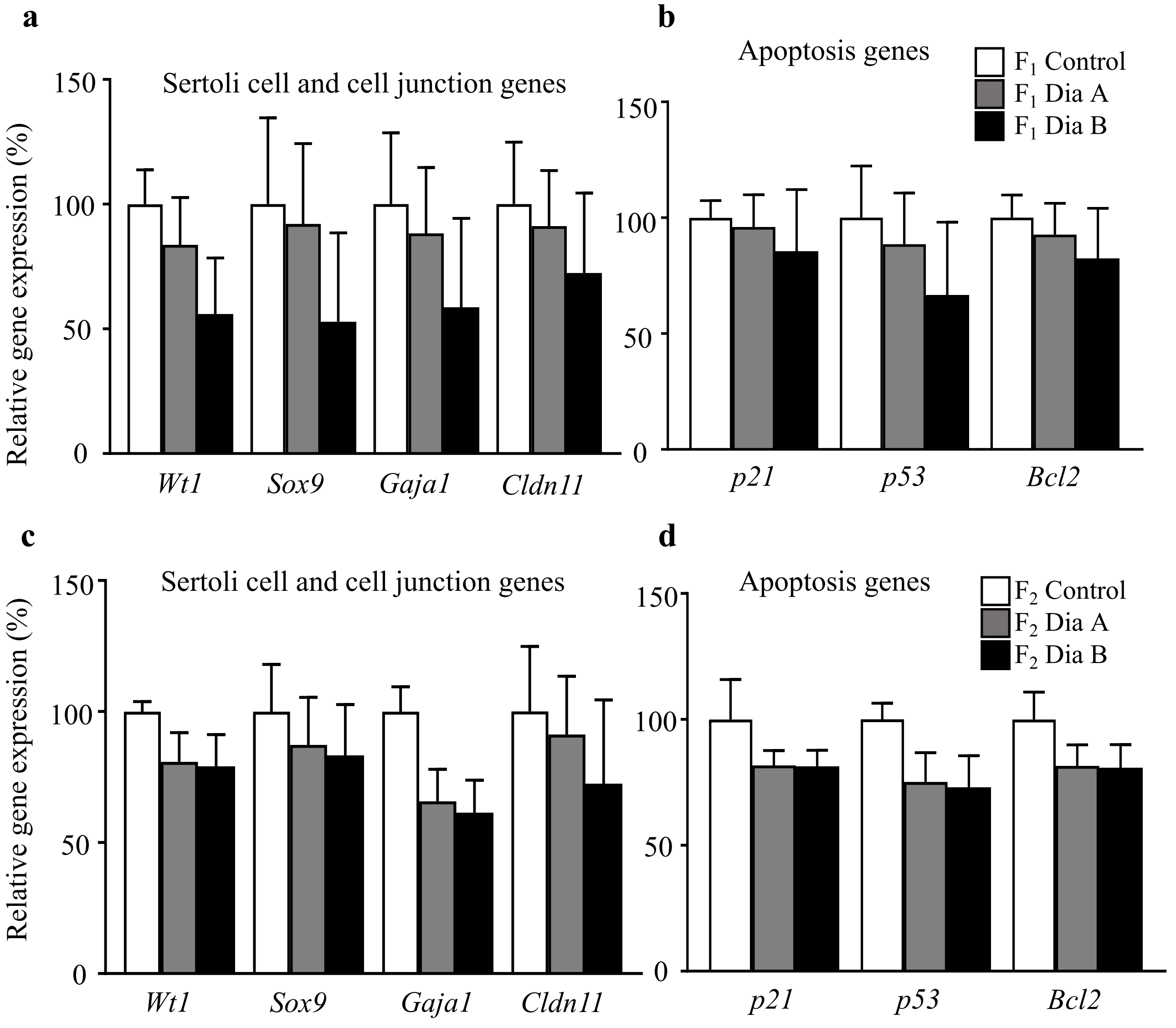

Supplementary Figure 1. Trangenerational effects of diabetes on testicular gene expression of the F_1 and F_2 male generations. The expression levels of genes encoding proteins associated with genes expressed in Sertoli cells: Wilms' tumour 1 (*Wt1*), SRY-box containing gene9 (*Sox9*), gap junction protein alpha 1 (*Gja1*) and claudin 11 (*Cldn11*) ; and apoptotic markers: cyclin-dependent kinase inhibitor 1A (*p21*), tumor protein p53 (*p53*), and B cell leukemia/lymphoma 2 (*Bcl2*) are shown (**a, b**) for the F_1 males (controls n = 8; diabetic group A n=10; diabetic group B n=5) and (**c, d**) for the F_2 males (controls n = 6; diabetic group A n=12; diabetic group B n=11). The graph illustrates the relative expression of the selected genes. The control group represents 100% and the percentage in the experimental groups represents the ratio of gene expression between the treated groups and the control group. The values are means ± SEM.

SUPPLEMENTARY DATA

Supplementary Table S1. List of antibodies with manufacturer reference number

Antibody	Reference	Company
mouse anti-VEGF-A	#sc-7269	Santa Cruz Biotechnology
rabbit anti-WT-1	#CA1026	Calbiochem
rabbit anti-pHH3	#06-570	Merck Millipore
rabbit anti-CX43	#C6219	Sigma
mouse anti-SCP3	#ab97672	Abcam
Alexa Fluor® 488	#115-545-146	Jackson Immuno Research Labs
Alexa Fluor® 594	#111-585-144	Jackson Immuno Research Labs

SUPPLEMENTARY DATA

Supplementary Table S2. List of primer sequences

Gene symbol	Gene name	RefSeq ID	Primer sequence	
Actb	Actin β	NM_007393.3	F	5' -CGGTTCCGATGCCCTGAGGCTCTT- 3'
			R	5' -CGTCACACTTCATGATGGAATTGA- 3'
Bcl-2	B-cell leucemia/lymphoma	NM_009741.3	F	5' -CAGGGAGATGTCACCCCTGGTGG- 3'
			R	5' -AGGCATCCCAGCCTCCGTTATCC- 3'
Cldn11	Claudin 11	NM_016674.4	F	5' -GGCGACATTAGTGGCCACAGCA- 3'
			R	5' -GCAGCGGCCCAGCCAGTAAA- 3'
Gja1	Gap junction alpha-1 protein	NM_010288.3	F	5' -TTTGGCGTGCCGGCTTCACTTT- 3'
			R	5' -CTCCGGCCGTGGAGTAGGCTT- 3'
Ppia	Peptidylprolyl isomerase A	NC_000077.5	F	5' -AGCTCTGAGCACTGGAGAGA- 3'
			R	5' -GCCAGGACCTGTATGCTTTA- 3'
Prm1	Protamine 1	NM_013637.4	F	5' -ACAGGTTGGCTGGCTCGACC- 3'
			R	5' -CGGCAGCATCGGTATCTGGCC- 3'
Prm2	Protamine 2	NM_008933.1	F	5' -CCAGGGGCCTGGACAAGACC- 3'
			R	5' -TCTGTGGTGGTGGTGGCCCC- 3'
p21	Cyclin-dependent kinase inhibitor 1	NM_007669.4	F	5' -CCGCCGCGGTGTCAGAGTCTA- 3'
			R	5' -CTGTGCGGAACAGGTCGGAC- 3'
p53	Tumor protein p53	NM_001127233.1	F	5' -ATGGCTTCCACCTGGGCTTCCTG- 3'
			R	5' -CCACAACTGCACAGGGCACGT- 3'
Sox9	SRY-box containing gene 9	NM_011448.4	F	5' -GCTGGAAGTCGGAGAGCCGAGA- 3'
			R	5' -AGAGAACGAAACCGGGGCCAC- 3'
Sycp1	Synaptonemal Complex Protein 1	NM_011516.2	F	5' -GCCCATGCTCGAACAG GTTGC- 3'
			R	5' -ACAGTCTGCTCATTGGCTCTGAA- 3'
Sycp3	Synaptonemal Complex Protein 3	NM_011517.2	F	5' -GGACAGCGACAGCTCACCGG- 3'
			R	5' -GGTGGCTTCCCAGATTTCCCAGA- 3'
Tnp1	Transition protein 1	NM_009407.2	F	5' -CCGAGCTCCTCACAAG GGCGT- 3'
			R	5' -CAGGGCAGAGCTCATTGCCGC- 3'
Tnp2	Transition protein 2	NM_013694.4	F	5' -CCTGCAAGACCCAGCCACCG- 3'
			R	5' -GTTTCCGCCTCCTGACGGCC- 3'
Wt1	Wilms Tumor 1	NM_144783.2	F	5' -GGCGCTTTGAGGGGTCCGAC- 3'
			R	5' -AAAGTGGGCGGAGCACCGAC- 3'
